# Russia-specific relative risks and their effects on the estimated alcohol-attributable burden of disease

**DOI:** 10.1186/s12889-015-1818-y

**Published:** 2015-05-10

**Authors:** Kevin D. Shield, Jürgen Rehm

**Affiliations:** Social and Epidemiological Research department, Centre for Addiction and Mental Health (CAMH), CAMH 33 Russell Street, Room T528, Toronto, ON M5S 2S1 Canada; WHO/PAHO Collaborating Centre in Addiction and Mental Health, CAMH, Toronto, Canada; Institute of Medical Science (IMS), University of Toronto, Toronto, Canada; Dalla Lana School of Public Health (DLSPH), University of Toronto, Toronto, Canada; Institute of Clinical Psychology and Psychotherapy, TU Dresden, Germany; Department of Psychiatry, University of Toronto, Toronto, Canada

**Keywords:** Alcohol, Russia, Eastern Europe, Relative risk, Risk factor, Burden of disease, Population-attributable fraction

## Abstract

**Background:**

Alcohol consumption is a major risk factor for the burden of disease globally. This burden is estimated using Relative Risk (RR) functions for alcohol from meta-analyses that use data from all countries; however, for Russia and surrounding countries, country-specific risk data may need to be used. The objective of this paper is to compare the estimated burden of alcohol consumption calculated using Russia-specific alcohol RRs with the estimated burden of alcohol consumption calculated using alcohol RRs from meta-analyses.

**Methods:**

Data for 2012 on drinking indicators were calculated based on the Global Information System on Alcohol and Health. Data for 2012 on mortality, Years of Life Lost, Years Lived with Disability, and Disability-Adjusted Life Years (DALYs) lost by cause were obtained by country from the World Health Organization. Alcohol Population-Attributable Fractions (PAFs) were calculated based on a risk modelling methodology from Russia. These PAFs were compared to PAFs calculated using methods applied for all other countries. The 95 % Uncertainty Intervals (UIs) for the alcohol PAFs were calculated using a Monte Carlo-like method.

**Results:**

Using Russia-specific alcohol RR functions, in Russia in 2012 alcohol caused an estimated 231,900 deaths (95 % UI: 185,600 to 278,200) (70,800 deaths among women and 161,100 deaths among men) and 13,295,000 DALYs lost (95 % UI: 11,242,000 to 15,348,000) (3,670,000 DALYs lost among women and 9,625,000 DALYs lost among men) among people 0 to 64 years of age. This compares to an estimated 165,600 deaths (95 % UI: 97,200 to 228,100) (29,700 deaths among women and 135,900 deaths among men) and 10,623,000 DALYs lost (95 % UI: 7,265,000 to 13,754,000) (1,783,000 DALYs lost among women and 8,840,000 DALYs lost among men) among people 0 to 64 years of age caused by alcohol when non-Russia-specific alcohol RRs were used.

**Conclusions:**

Results indicate that if the Russia-specific RRs are used when estimating the health burden attributable to alcohol consumption in Russia, then the total estimated burden will be more than if RRs from meta-analyses are used. Furthermore, additional research is needed to understand which aspects of the Russian style of drinking cause the most harm.

**Electronic supplementary material:**

The online version of this article (doi:10.1186/s12889-015-1818-y) contains supplementary material, which is available to authorized users.

## Background

Alcohol has been shown to cause a considerable burden of disease [1, 2]. Historically, estimates of the alcohol-attributable burden have been based on a conceptual Population-Attributable Fraction (PAF) model that combines Relative Risks (RRs) taken from meta-analyses [3, 4] with alcohol exposure estimates [5, 6]. However, estimates based on this model may not accurately reflect the true proportion of disease caused by alcohol for countries with extreme and varying drinking patterns. For example, consumption patterns found in Russia include long bouts of continuous heavy drinking alternating with days of abstinence [7–9], which negatively impact on ischemic heart disease (IHD), ischemic stroke and injuries [10–13].

Historically, alcohol has had a great impact on health in Russia [14, 15]. Furthermore, the overall health of the Russian population is considered poor by Western European standards, and Russians, and in particular Russian men, have a low life expectancy: the 2012 life expectancy was 63 years for men and 75 years for women [16]. Alcohol has been found to be the most important factor responsible for the low life expectancy for men [15, 17–20]. Indeed, when alcohol consumption dropped markedly as a result of the Gorbachev reforms, mortality rates dropped by 11.4 % [18, 21], exemplifying the negative impact of alcohol consumption on the health of the Russian population. Less dramatic, but still visible, were the more recent impacts of alcohol policy on mortality and life expectancy [22].

The production and consumption of alcohol is heavily ingrained in Russian society [7, 23]. Russia and other Eastern European countries consume more alcohol than any other countries in the world [2, 24]. Additionally, Russians consume alcohol in the comparatively most harmful manner, namely irregular very heavy drinking, mostly outside of meals and associated with intoxication (for associations between these patterns of drinking and health see [4, 8, 25, 26]). Russia and surrounding Eastern European countries received pattern of drinking scores (a measure of the way alcohol is consumed, rather than the amount of alcohol that is consumed) of 5, indicating the most detrimental way of drinking [2]. As people in these countries consume alcohol in a very detrimental manner, the application of global RRs may be problematic for these countries.

Accordingly, the 2010 Global Burden of Disease (GBD) study used Russia-specific RRs based on average consumption of alcohol data obtained from a large cohort study in Russia [14] in order to model the burden of disease attributable to alcohol in Russia and countries with similar drinking patterns [1]. To date, there has been no systematic comparison of the resulting estimated burden of disease attributable to alcohol consumption using Russia-specific RRs compared to using the RRs from meta-analyses that combined risk information from multiple countries; however, the most recent World Health Organization (WHO) alcohol burden of disease study for 2012 [2] provides the data to make this comparison. This paper: (i) presents a method used to calculate the alcohol PAFs for Russia based on Russia-specific RR functions, (ii) uses this method to estimate and describe the burden of disease attributable to alcohol in Russia, and (iii) compares this estimated burden of disease to the estimated burden calculated using RRs that combined data from all studies.

## Methods

The estimates for the burden of disease attributable to alcohol consumption are limited by inaccuracies in the cause of death [27–29], and this error increases for the elderly as pathologies may become more complicated [30, 31]. Accordingly, this study was limited to Russians 0 to 64 years of age.

### Estimating the alcohol population-attributable fractions

A PAF is the proportion of mortality or morbidity that would not have occurred if the exposure of every person in the population to a risk factor were at the theoretical minimum risk exposure level [1, 32, 33]. Even though protective effects of alcohol can be observed for ischemic diseases and diabetes for people who consume little alcohol and do not engage in Heavy Episodic Drinking (HED) [10, 34, 35], the PAFs for alcohol use the theoretical minimum risk exposure of lifetime abstention and are calculated for most chronic diseases (except IHD and injuries) (see appendix for methodology) using Formula 1.

(Formula 1)

$$ PAF=\frac{P_{abs}+{P}_{former}R{R}_{former}+{\displaystyle {\int}_{>0}^{150}}{P}_{current}(x)R{R}_{current}(x)dx-1}{P_{abs}+{P}_{former}R{R}_{former}+{\displaystyle {\int}_{>0}^{150}}{P}_{current}(x)R{R}_{current}(x)dx} $$

where P_abs_ is the prevalence of lifetime abstainers, P_former_ is the prevalence of former drinkers, RR_former_ is the RR for former drinkers, P_current_ is the prevalence of current drinkers who consume on average x grams of alcohol, and RR_current_ is the RR given an average daily consumption of x grams of alcohol. The full methods for the calculation of the alcohol PAFs are outlined in Additional file 1 (HED is used in the estimation of the PAFs for injuries and IHD when using the 2010 GBD and 2012 WHO alcohol burden of disease study methodologies).

### Mortality, morbidity and population data

Data for 2012 on mortality (deaths and Years of Life Lost (YLL)), morbidity (Years Lived with Disability (YLD)), and Disability-Adjusted Life Years (DALYs) lost (a combination of YLL and YLD) were obtained from the WHO [2]. Population data by age and sex were obtained from the United Nations population division [36].

### Indicators of alcohol consumption

Data for the drinking status indicators for 2012, namely lifetime abstainers (those who have not consumed at least one standard drink of alcohol within their lifetime), former drinkers (those who have consumed at least one standard drink of alcohol, but have not done so in the past year), and current drinkers (those who have consumed at least one standard drink of alcohol in the past year), the prevalence of HED, the relative amount of alcohol consumed among drinkers differentiated by sex and age groups (15 to 34, 35 to 64 and 65 years of age and older) were obtained from the Global Information System on Alcohol and Health, which inputs country-specific data (for Russia: [37]) into a regression equation with the following variables: alcohol consumption (from survey data and *per capita* data), year, Gross Domestic Product (adjusted for purchase power parity), population age structure and the prevalence of people who identify themselves as Muslims (see [2] for more information). This regression method adjusted for inconsistencies between country-specific data for 2012 and data from surveys of the same nature conducted in similar countries, thereby bringing data on the prevalence of HED and the prevalence of different drinking statuses in line with those of other similar countries. This is especially important for Russia, as the data outlined in [37] have been criticized for being substantial underestimates of the prevalence of HED [38, 39].

These survey-based data were triangulated with data on recorded and unrecorded alcohol *per capita* consumption (estimated based on data obtained from the government of Russia [40] and Nemtsov [21]). Adult *per capita* consumption of alcohol is considered the best measure of alcohol consumption as surveys often underestimate alcohol consumption [41, 42]. This is especially important for Russia, as the data outlined in [37] have also been criticized for being substantial underestimates the volume of alcohol consumed [38, 39]. The distribution of alcohol consumption among current drinkers by age and sex for Russia in 2012 was estimated using methodology developed by Rehm and colleagues and Kehoe and colleagues [6, 43]. (See Additional file 1 for more details).

### Risk relations

For this study we modelled the burden caused by alcohol consumption using RRs specific to Russia (which were based on average consumption) and RRs based on meta-analyses. The RR functions used for Russia were obtained from the study by Zaridze and colleagues [14] for tuberculosis, acute and chronic pancreatitis, pneumonia, stroke (ischemic and hemorrhagic), IHD, liver cirrhosis, suicide, homicide and assaults, injuries of undetermined intent, and other injuries. The RRs for Russia were based on the following weekly alcohol consumption categories (the equivalent daily alcohol consumption figures in pure alcohol are given in parentheses): 1) greater than one half but less than one bottle (>12.68 and < 25.36 grams (g) of pure alcohol per day (the lower bound was not used in our calculations)), 2) one to less than three bottles (equivalent to ≥ 25.36 grams to < 76.08 grams of pure alcohol per day), and 3) three or more bottles (≥ 76.08 grams of pure alcohol per day). The reference category for these RRs was current drinkers who consume less than one half of a bottle of vodka on a weekly basis; to adjust for this we applied the RR for people who consume greater than one half but less than one bottle of vodka a week to people who consume less than one half of a bottle of vodka per week. These Russia-specific categorical RRs were then transformed into a piecewise function and were then used in Formula 1 as RR_current_ to estimate the alcohol PAFs. Data on the RRs for former drinkers were obtained from the meta-analyses used for other countries and data on the RRs for other diseases were obtained from other sources (see Additional file 2). See Additional file 3 for a comparison of the meta-analysis RRs with the Russia-specific RRs.

The study by Zaridze and colleagues calculated data on 48,557 adult deaths for people 15 to 74 years of age, and obtained details of alcohol consumption from proxy information provided by family members. The RRs controlled for age, smoking (“did smoke” or “did not smoke”) and city of residence [14].

### Estimating the 95 % uncertainty intervals for the PAFs

The 95 % Uncertainty Intervals (UIs) were estimated using a Monte Carlo-type approach [44]. To estimate the 95 % UIs for each alcohol PAF, we used a set of 2,000 generated lowest level parameters; these sets of parameters were then used to calculate 2,000 alcohol PAFs. The calculated variances of the resulting 2,000 alcohol PAFs were used to estimate the 95 % UIs.

All statistical analyses and modelling were performed using R version 3.1.0 [45].

## Results

In Russia in 2012, the adult *per capita* consumption of alcohol was 14.8 litres of pure alcohol (23.4 litres of pure alcohol per adult male and 7.6 litres of pure alcohol per adult female), with recorded adult *per capita* consumption of alcohol being 11.2 litres of pure alcohol and unrecorded adult *per capita* consumption of pure alcohol being 3.6 litres of alcohol. Table 1 outlines alcohol consumption in Russia for 2012. In that year, more men than women were current drinkers and engaged in HED. Furthermore, more men than women were heavy consumers of alcohol (a daily consumption of greater than 76.08 grams of pure alcohol per day).

**Table 1:** Alcohol consumption characteristics of the Russian population in 2012

Gender	Alcohol consumption		Age			
			15 to 34 years of age	35 to 64 years of age	65 years of age and older	Total (15 years of age and older)
Men	Lifetime abstainers		6.6 %	5.4 %	11.5 %	6.5 %
	Former drinkers		15.0 %	20.4 %	24.2 %	18.7 %
	Current drinkers		78.4 %	74.2 %	64.3 %	74.8 %
		<12.68 g/day*	23.2 %	22.8 %	22.8 %	22.9 %
		>12.68 g/day and < 25.36 g/day	12.9 %	12.5 %	11.7 %	12.5 %
		≥25.36 g/day to < 76.08 g/day	27.5 %	25.9 %	21.5 %	26.0 %
		≥76.08 g/day	14.9 %	13.1 %	8.3 %	13.3 %
	Heavy episodic drinkers**		31.2 %	29.5 %	25.6 %	29.8 %
Women	Lifetime abstainers		14.7 %	16.0 %	32.5 %	18.8 %
	Former drinkers		18.4 %	18.5 %	24.7 %	19.7 %
	Current drinkers		66.9 %	65.4 %	42.8 %	61.5 %
		<12.68 g/day	32.7 %	33.8 %	30.2 %	32.8 %
		>12.68 g/day and < 25.36 g/day	12.6 %	12.5 %	7.5 %	11.6 %
		≥25.36 g/day to < 76.08 g/day	17.5 %	16.0 %	5.0 %	14.4 %
		≥76.08 g/day	4.1 %	3.1 %	0.2 %	2.9 %
	Heavy episodic drinkers**		11.1 %	10.8 %	7.1 %	10.2 %
Total	Lifetime abstainers		10.6 %	11.1 %	25.9 %	13.2 %
	Former drinkers		16.7 %	19.4 %	24.5 %	19.2 %
	Current drinkers		72.7 %	69.5 %	49.6 %	67.6 %
		<12.68 g/day	27.9 %	28.7 %	27.8 %	28.3 %
		>12.68 g/day and < 25.36 g/day	12.7 %	12.5 %	8.8 %	12.0 %
		≥25.36 g/day to < 76.08 g/day	22.5 %	20.5 %	10.2 %	19.7 %
		≥76.08 g/day	9.5 %	7.7 %	2.8 %	7.6 %
	Heavy episodic drinkers**		21.2 %	19.4 %	12.9 %	19.1 %

*Measured in grams of pure alcohol per day

**Monthly consumption of at least 60 grams of pure alcohol on at least one drinking occasion

In 2012, for people 0 to 64 years of age, using Russia-specific alcohol RRs, alcohol caused an estimated 231,900 (95 % UI: 185,600 to 278,200) deaths in Russia (70,800 deaths among women and 161,100 deaths among men), and 13,295,000 DALYs were lost (95 % UI: 11,242,000 to 15,348,000) (3,670,000 DALYs lost among women and 9,625,000 DALYs lost among men) due to alcohol consumption. Adjusting these figures for population size results in 187 deaths per 100,000 people (118 and 251 deaths per 100,000 women and men respectively) and 10,694 DALYs lost per 100,000 people (6,091 and 15,023 DALYs lost per 100,000 women and men respectively).

This burden represents 29.4 % of all deaths for this age group (31.2 % and 28.7 % of all deaths for women and men respectively in this age group), and 27.5 % of all DALYs lost (21.9 % of all DALYs lost for women and 30.5 % of all DALYs lost for men). See Tables 2 and 3 for the burden of alcohol consumption by cause for deaths and DALYs lost respectively based on Russia-specific alcohol RRs.

**Table 2:** Burden of deaths attributable to alcohol consumption in Russia for people 0 to 64 years of age in 2012 based on Russia-specific alcohol RR functions

Cause*			Women		Men		Total	
			Deaths	% of all deaths AA	Deaths	% of all deaths AA	Deaths	% of all deaths AA
Total			70,800	31.2 %	161,100	28.7 %	231,900	29.4 %
	Communicable, maternal, neonatal and nutritional disorders		3,510	13.4 %	12,620	16.8%	16,130	15.9 %
		HIV/AIDS and tuberculosis	1,470	8.5 %	7,950	14.0 %	9,420	12.7 %
		Lower respiratory infections	1,960	42.8 %	4,550	35.3 %	6,510	37.2 %
		Neonatal conditions	80	3.3 %	120	3.4 %	200	3.3 %
	Non-communicable diseases		51,810	29.9 %	84,850	22.8 %	136,660	25.1 %
		Neoplasms	3,810	6.8 %	8,120	10.1 %	11,930	8.8 %
		Diabetes mellitus	−280	−13.9 %	−10	−0.5 %	−290	−7.8 %
		Cirrhosis of the liver	11,750	76.2 %	11,750	50.0 %	23,500	60.4 %
		Digestive diseases (except cirrhosis)	970	16.1 %	3,210	18.1 %	4,180	17.6 %
		Neurological disorders	90	4.5 %	210	8.4 %	300	6.7 %
		Mental and behavioural disorders	3,580	67.1 %	14,850	71.3 %	18,430	70.4 %
		Cardiovascular and circulatory diseases	31,900	42.0 %	46,720	22.4 %	78,620	27.7 %
	Injuries		15,510	55.3 %	63,600	55.5 %	79,110	55.5 %
		Transport injuries	2,120	41.0 %	8,230	47.7 %	10,350	46.1 %
		Unintentional non-transport injuries**	8,990	57.1 %	35,380	55.8 %	44,370	56.0 %
		Self-harm and personal violence***	4,400	62.7 %	20,000	60.7 %	24,400	61.0 %

*See Additional file 2 for ICD categories included in each cause

**Includes poisonings, falls, fires, drowning and other unintentional injuries

***Includes self-inflicted injuries and homicide

**Table 3:** Burden of Disability-Adjusted Life Years lost attributable to alcohol consumption in Russia for people 0 to 64 years of age in 2012 based on Russia-specific alcohol RR functions

Cause*			Women		Men		Total	
			DALYs	% of all DALYs AA	DALYs	% of all DALYs AA	DALYs	% of all DALYs AA
Total			3,670,000	21.9 %	9,625,000	30.5 %	13,295,000	27.5 %
	Communicable, maternal, neonatal and nutritional disorders		183,200	8.5 %	620,100	14.3 %	803,300	12.4 %
		HIV/AIDS and tuberculosis	87,600	9.3 %	434,600	15.0 %	522,200	13.6 %
		Lower respiratory infections	86,700	29.0 %	172,800	28.9 %	259,500	28.9 %
		Neonatal conditions	8,900	3.1 %	12,600	3.2 %	21,500	3.2 %
	Non-communicable diseases		2,541,000	19.8 %	5,487,700	26.4 %	8,028,700	23.9 %
		Neoplasms	145,400	6.8 %	291,800	10.0 %	437,200	8.7 %
		Diabetes mellitus	−45,700	−13.7 %	−1,500	−0.5 %	−47,200	−7.8 %
		Cirrhosis of the liver	494,900	76.1 %	510,000	49.8 %	1,004,900	60.0 %
		Digestive diseases (except cirrhosis)	43,600	15.1 %	152,900	19.0 %	196,500	17.9 %
		Neurological disorders	16,600	3.2 %	32,400	8.3 %	49,000	5.4 %
		Mental and behavioural disorders	637,500	22.3 %	2,698,200	59.7 %	3,335,700	45.2%
		Cardiovascular and circulatory diseases	1,248,800	41.1 %	1,803,700	22.1 %	3,052,500	27.2 %
	Injuries		946,000	53.5 %	3,517,100	54.4 %	4,463,100	54.2 %
		Transport injuries	157,100	41.3 %	552,400	47.5 %	709,500	46.0 %
		Unintentional non-transport injuries**	555,700	55.4 %	1,890,700	54.6 %	2,446,400	54.8 %
		Self-harm and personal violence***	233,200	61.5 %	1,074,000	60.2 %	1,307,200	60.4 %

*See Additional file 2 for ICD categories included in each cause

**Includes poisonings, falls, fires, drowning and other unintentional injuries

***Includes self-inflicted injuries and homicide

Furthermore, in Russia in 2012, for people 0 to 64 years of age, alcohol-attributable communicable, maternal, neonatal and nutritional disorders contributed to 7.0 % of all alcohol-attributable deaths and 6.0 % of all alcohol-attributable DALYs lost. Alcohol-attributable non-communicable diseases contributed to 58.9 % of all alcohol-attributable deaths and 60.4 % of all alcohol-attributable DALYs lost, and alcohol-attributable injuries contributed to 34.1 % of all alcohol-attributable deaths and 33.6 % of all alcohol-attributable DALYs lost. Self-harm and personal violence as well as mental and behavioural disorders (the burden of this category stemmed mainly from alcohol use disorders) were impacted the most by alcohol; 61.0 % of all deaths and 60.4 % of all DALYs lost caused by self-harm and personal violence and 70.5 % of all deaths and 42.8 % of all DALYs lost caused by mental and behavioural disorders were attributable to alcohol consumption. Transport injuries, unintentional non-transport injuries, cirrhosis of the liver, cardiovascular and circulatory diseases, and lower respiratory infections were also greatly impacted by alcohol.

Compared to the 231,900 deaths based on country specific data (see above), an estimated 165,600 deaths (95 % UI: 97,200 to 228,100) (29,700 deaths among women and 135,900 deaths among men) were caused by alcohol based on using the alcohol RR functions from the meta-analyses used for other countries. These differences in the estimated numbers of deaths stem primarily from differences in the number of deaths estimated to be caused by injuries, ischemic strokes and IHD attributable to alcohol; there was little observed difference in the mortality burdens for pneumonia, hemorrhagic and other non-ischemic strokes, and cirrhosis of the liver attributable to alcohol. See Table 4 for the number of deaths and DALYs lost by cause attributable to alcohol for men and women using Russia-specific alcohol RR functions and using general alcohol RR functions. See Additional file 4 for the number of YLL and YLD by cause attributable to alcohol consumption for men and women using Russia-specific alcohol RR functions and using general alcohol RR functions.

**Table 4:** Deaths and DALYs lost attributable to alcohol consumption in Russia in 2012 using Russia-specific alcohol RR functions and general population alcohol RR functions for people 0 to 64 years of age

			Women		Men		Total	
	Cause*		Deaths	DALYs	Deaths	DALYs	Deaths	DALYs
Russia-specific alcohol RRs								
	Communicable, maternal, neonatal and nutritional disorders							
		Lower respiratory infection	1,960	86,700	4,550	173,000	6,510	259,700
	Non-communicable diseases							
		Ischemic heart disease	25,520	1,009,600	38,870	1,507,000	64,390	2,516,600
		Ischemic stroke	2,430	86,500	3,260	116,000	5,690	202,500
		Hemorrhagic and other non-ischemic strokes	3,400	129,600	3,520	137,000	6,920	266,600
		Cirrhosis of the liver	11,750	494,900	11,750	510,000	23,500	1,004,900
		Acute and chronic pancreatitis	970	43,600	3,210	153,000	4,180	196,600
	Injuries							
		Transport injuries	2,120	157,100	8,230	552,000	10,350	709,100
		Unintentional injuries (other than transport injuries)**	8,990	555,700	35,380	1,891,000	44,370	2,446,700
		Self-harm and personal violence***	4,400	233,200	20,000	1,074,000	24,400	1,307,200
	Total		70,800	3,670,000	161,100	9,625,000	231,900	13,295,000
	Total (per 100,000 people)		118	6,091	251	15,023	187	10,694
Non-Russia-specific alcohol RRs								
	Communicable, maternal, neonatal and nutritional disorders							
		Lower respiratory infection	410	18,500	1,940	74,200	2,350	92,700
	Non-communicable diseases							
		Ischemic heart disease	130	−21,900	−5,570	−227,600	−5,440	−249,500
		Ischemic stroke	−300	−10,300	1,500	52,900	1,200	42,600
		Hemorrhagic and other non-ischemic strokes	3,880	146,300	5,900	230,600	9,780	376,900
		Cirrhosis of the liver	10,070	425,800	18,680	811,900	28,750	1,237,700
		Acute and chronic pancreatitis	360	16,200	2,920	139,600	3,280	155,800
	Injuries							
		Transport injuries	1,440	96,900	15,110	961,200	16,550	1,058,100
		Unintentional injuries (other than transport injuries)**	2,960	161,500	40,930	2,085,700	43,890	2,247,200
		Self-harm and personal violence***	1,420	76,800	22,160	1,199,100	23,580	1,275,900
	Total		29,700	1,783,000	135,900	8,840,000	165,600	10,623,000
	Total (per 100,000 people)		49	2,959	212	13,798	133	8,545

*See Additional file 2 for ICD categories included in each cause

**Includes poisonings, falls, fires, drowning and other unintentional injuries

***Includes self-inflicted injuries and homicide

When using the alcohol RR functions from the meta-analyses, 10,623,000 DALYs (95 % UI: 7,265,000 to 13,754,000) were lost (1,783,000 DALYs lost among women and 8,840,000 DALYs lost among men) for people 0 to 64 years of age. This compares to an estimated 13,295,000 DALYs lost (95 % UI: 11,242,000 to 15,348,000) (3,670,000 DALYs lost among women and 9,625,000 DALYs lost among men) caused by alcohol consumption for people 0 to 64 years of age when using the Russia-specific alcohol RR functions. These differences in the numbers of DALYs lost stem primarily from differences in the number of DALYs lost caused by injuries, ischemic strokes and IHD attributable to alcohol consumption. See Additional file 5 for the burden of disease modelled for all ages.

## Discussion

This study presents data that suggests that modelling the burden of alcohol consumption using RRs based on meta-analyses from all countries may not be appropriate, especially for diseases and injuries where the risk is determined in part by drinking patterns and other factors such as drink-driving laws [12, 13]. Specifically, this study found that the alcohol-attributable burden of transport injuries was moderately higher when non-Russia-specific RRs were used and that alcohol-attributable ischemic disease was markedly underestimated when non-Russia-specific RRs were used. This study also found that the alcohol-attributable burdens of acute and chronic pancreatitis, unintentional injuries (other than transport injuries), self-harm and personal violence were lower when using non-Russia-specific RRs, and that the burdens of hemorrhagic and other non-ischemic strokes, and cirrhosis of the liver were higher when using non-Russia-specific RRs (see Additional file 3 for a comparison of the Russian RRs with categorically transformed RRs from meta-analyses).

Differences in the estimated alcohol-attributable burden using the Russia-specific RRs versus the general RRs were most evident for women, as the protective effect on IHD is more pronounced among women [10]). However, the differences in the estimated alcohol-attributable burden using the Russia-specific RRs versus the general RRs may be diminished if the studies which provide new evidence of the detrimental effects of alcohol on IHD (which have only been recently published) were used in modeling general RRs for IHD [46, 47]. Moreover, results of Mendelian randomization studies on alcohol and IHD have put into question the existence of a beneficial effect (see [48], and the subsequent discussion [49, 50]).

The burden of transport injuries caused by alcohol was less when calculated using the Russia-specific RR functions compared to when the general alcohol RR functions were used. This may be due to the different methodologies used in the two estimates (one based on average consumption and the other based on average and heavy drinking occasions).

When restricting our findings to people 15 to 64 years of age, this study found that 32.4 and 29.3 % of all deaths among women and men respectively were attributable to alcohol consumption. These findings are in contrast to those reported by Leon and colleagues [8] who found that 43 % of all premature deaths among men 25 to 54 years of age were attributable to hazardous drinking as defined by non-beverage alcohol consumption and/or problem drinking, and to Zaridze and colleagues’ findings that 52 % of all deaths among people 15 to 54 years of age were attributable to alcohol consumption [14]. One explanation for part of the observed differences is that in our study alcohol poisoning deaths (which constituted a large percentage of excess deaths in Leon and colleagues’ and Zaridze and colleagues’ studies) could not be modelled using a specific category RR, as data on alcohol poisonings were not available as a separate injury category. Furthermore, the percentage of all deaths attributable to alcohol consumption was greater for women than for men, even though men experienced more deaths attributable to alcohol consumption. The factors that caused women to have such a high relative burden of alcohol consumption should be investigated in future research.

The current results underline the necessity to check on the applicability of global RR functions for individual countries. For Russia and some surrounding Eastern European countries, RR functions may underestimate true risk because of drinking patterns. Additionally, for other countries, prevalences of people with genotypes that influence the risk of alcohol consumption may also lead to incorrect burden estimates when global RR functions are applied [51].

### Limitations

There are limitations to the Russia-specific RRs from Zaridze and colleagues’ study. First, these RRs are step functions, in contrast to continuous risk functions which are used for other countries. Second, the measurement of alcohol consumption for Zaridze and colleagues’ study was by proxy (in 2001–2005) and was measured years after the mortality of the individuals (1990–2001). Third, the data for Russia-specific RRs are based on one large study and, as a consequence, some RRs were not significant [14] and, thus, were not used. Fourth, the Russia-specific RRs are solely for mortality and not morbidity, whereas meta-analyses have recently started to assess morbidity separately [3]. Fifth, the Russia-specific alcohol RRs use only average alcohol consumption and use the reference category of ever-drinkers with weekly consumption of less than one half bottle of vodka. The use of this reference category may lead to a minor underestimation of the risk of mortality from alcohol-related diseases as we treated this category as lifetime abstainers within the alcohol PAF. The use of the RR for consumption of between less than one bottle but greater than one half of a bottle of vodka a week to all current drinkers that consumed less than one bottle of vodka a week should not cause an overestimation of the alcohol-attributable burden of IHD, as the protective effects of alcohol on IHD occur at moderate alcohol consumption [10]. Finally, Russia-specific RRs were measured for people who died from 1990 to 2001. This timeframe includes the Russian mortality crisis (from 1990 to 1994) where Russia experienced a 40 % increase in the crude mortality rate and alcohol poisoning also increased [52, 53]. Therefore, the RRs may not reflect the current situation in Russia as some 100 % alcohol-attributable mortalities, such as alcohol poisonings, have since decreased [53].

The estimates for the burden of disease attributable to alcohol consumption have general limitations. First, mortality and morbidity data contain inaccuracies, even when obtained from the most reliable sources [27–29], with the measurement of the burden of morbidity being commonly subject to more error than the burden of mortality [54]. For Russia and similar countries, the estimates of YLD are very provisional and uncertain in nature, making estimates of the alcohol-attributable DALYs lost for Russia presented in this study less accurate than the estimates presented for alcohol-attributable mortality [55, 56]. Specifically, the databases which result from the collection of morbidity data from polyclinics and hospitals (the sources of most morbidity data) are problematic in terms of their diagnostic accuracy [55]. Additionally, even data collected in Russia in a systematic manner and on a large scale (such as cancer registries) have problems with accuracy and detail [55]. Second, the estimates presented are based on RR functions that were adjusted for age and smoking status, and in some cases for a variety of other risk factors, which may introduce bias [33, 57, 58]. Third, the estimates for alcohol consumption are cross-sectional, and past alcohol consumption would be a better risk measure for chronic diseases such as cancer and liver cirrhosis which develop over time [3]; however, for diseases such as liver cirrhosis, changes in alcohol consumption have been observed to have an immediate impact on mortality [18, 59].

## Conclusions

Russia-specific RRs produce different estimates of the alcohol-attributable burden of disease when compared to estimates obtained using the RRs from meta-analyses of global data. Further investigation into the reasons for these differences should be undertaken, as country-specific RRs may be needed in cases where countries have very detrimental drinking patterns.

## Additional files

Additional file 1:
**Methods used to model alcohol Population-Attributable Fractions and methods used to model the distribution of alcohol consumption.**
Additional file 2:
**Sources of Relative Risk functions and of Relative Risk function plots.**
Additional file 3:
**Comparison of the RR functions from meta-analyses to those obtained from Zaridze et al., [** 14 **].**
Additional file 4:
**YLL and YLD attributable to alcohol consumption in Russia in 2012 using Russia-specific alcohol RR functions and general population alcohol RR functions for people 0 to 64 years of age.**
Additional file 5:
**Deaths, YLL, YLD and DALYs lost attributable to alcohol consumption using Russia-specific alcohol RR functions and general population alcohol RR functions for people of all ages.**

## Abbreviations

BAC: Blood alcohol content
DALYs: Disability-adjusted life years
GBD: Global burden of disease
HED: Heavy episodic drinking
IHD: Ischemic heart disease
PAFs: Population-attributable fractions
YLL: Years of life lost
RR: Relative risk
UIs: Uncertainty intervals
WHO: World health organization
YLD: Years lived with disability
