# Supplementary material for: Russia-specific relative risks and their effects on the estimated alcohol-attributable burden of disease
Source: BMC Public Health. 2015 May 10;15:482. doi: 10.1186/s12889-015-1818-y (PMC4489203; doi:10.1186/s12889-015-1818-y)
Supplement: Additional file 1: — Methods used to model alcohol Population-Attributable Fractions and methods used to model the distribution of alcohol consumption. [file 12889_2015_1818_MOESM1_ESM.docx]

**Additional file 1.** Methods used to model alcohol Population Attributable Fractions and methods used to model the distribution of alcohol consumption.

**Deriving Alcohol-Attributable Fractions based on exposure and Relative Risks**

The number of deaths, Potential Years of Life Lost (PYLL), Years Lived with Disability (YLD) and Disability Adjusted Life Years (DALYs) attributable to alcohol consumption (i.e. the number of deaths that would not have occurred if everyone was a lifetime abstainer) is calculated using an alcohol Population Attributable Fraction (PAF) [[1](#_ENREF_1), [2](#_ENREF_2)].

To calculate the number of deaths, PYLL, YLD, DALYs attributable to alcohol consumption we: 1) calculated the country-, age-, and sex-specific alcohol PAFs, and then 2) applied these alcohol PAFs to the corresponding mortality, PYLL, YLD and DALYs lost data.

**Step 1: Calculation of the alcohol PAFs by country, age, and sex**

***Definition of age categories***

Three age categories were used in our analysis so as to be comparable to other Global Burden of Disease (GBD) studies: 15-34, 35-64 and 65 or older [[3](#_ENREF_3)].

***Sources for modelling risk relations***

Sources for Relative Risk (RR) functions are outlined in the main text for Russia specific RRs and Web Appendix 2 for general RRs. These RRs are based on meta-analyses that report a categorical RR continuous RR function by alcohol consumption exposure (see [[4](#_ENREF_4)] for an overview of the relationship between alcohol and various diseases, conditions and injuries).

***Alcohol PAFs for chronic and infectious diseases, except ischemic heart disease***

The alcohol PAFs for all Russia specific calculations and for all non-Russia specific chronic and infectious diseases (except ischemic heart disease), were calculated as follows:

$$PAF=\frac{P_{abs}+P_{former}{RR}_{former}+\int_{>0}^{150} P_{current}\left( x \right){RR}_{current}\left( x \right)dx-1}{P_{abs}+P_{former}{RR}_{former}+\int_{>0}^{150} P_{current}(x){RR}_{current}(x)dx}$$

where P_abs_ is the prevalence of lifetime abstainers, P_former_ is the prevalence of former drinkers, RR_former_ is the RR for former drinkers, P_current_ is the prevalence of current drinkers who consume on average x grams of alcohol, and RR_current_ is the RR given an average daily consumption of x grams of alcohol.

***Alcohol PAFs for ischemic heart disease***

Average volume of alcohol consumption and patterns of drinking are associated with the risk of ischemic heart disease [[5](#_ENREF_5), [6](#_ENREF_6)]. To model the risk of ischemic heart disease, we used an RR based on average volume of consumption ([[7](#_ENREF_7)]; see also [[8](#_ENREF_8)], or [[9](#_ENREF_9)] for similar results) for all individuals who did not engage in heavy drinking (see Table 3 above). For people with at least one heavy drinking occasion per month (see Table 3 above), we assumed no cardio-protective effect [[10](#_ENREF_10)] (i.e. a RR of 1).

***Estimating the alcohol PAFs for people who sustain injuries due to other people’s drinking, including low birth weight***

To estimate the mortality caused by low birth weight attributable to alcohol consumption, we modelled the alcohol consumption of pregnant women who drank the same amount of alcohol as pre-pregnancy and the alcohol consumption of pregnant women who drank less. The alcohol PAFs for low birth weight were calculated as follows:

$$PAF=\frac{P_{abs}+\int_{>0}^{150} P_{less}\left( x \right)RR\left( x \right)dx+ \int_{>0}^{150} P_{same}\left( x \right)RR\left( x \right)dx-1}{P_{abs}+\int_{>0}^{150} P_{less}\left( x \right)RR\left( x \right)dx+ \int_{>0}^{150} P_{same}\left( x \right)RR\left( x \right)dx}$$

where P_abs_ is the proportion of women who abstained from consuming alcohol while pregnant, P_same_ represents the proportion of pregnant women who consumed the same amount of alcohol as pre-pregnancy, and P_less_ represents the proportion of pregnant women who consumed less alcohol than pre-pregnancy.

***Alcohol PAFs for injuries***

*Estimating the alcohol PAFs for harms caused to oneself*

To estimate the morbidity from injuries that are attributable to alcohol consumption, we took into account two dimensions of alcohol consumption:

1. binge drinking (both the number of occasions and the amount consumed per occasion), and
2. alcohol consumption (on non-binge days).

We also modelled the time at risk for injury based on alcohol metabolism rates for men and women, according to methods outlined by Taylor and colleagues [[11](#_ENREF_11)].

The alcohol PAFs for injuries attributable to alcohol consumption were calculated as follows:

**

where P_abs_ is the prevalence of current abstainers, and P_current(binge)_ and P_current(non-binge)_ are the prevalence of current drinkers who engage in binge drinking and the prevalence of current drinkers who do not engage in binge drinking respectively. The RRs for current drinkers who engage in binge drinking, and current drinkers who do not engage in binge drinking were calculated as follows:

$RR_{\mathrm{current}\left( non-binge \right)}=\left( RR_{\mathrm{average}}-1 \right)*P_{\mathrm{nonbingedays}}+$ $RR_{\mathrm{current}\left( non-binge \right)}=\left( RR_{\mathrm{average}}-1 \right)*P_{\mathrm{nonbingedays}}+$1

and:

$$RR_{current\left( binge \right)}=\left( RR_{average}-1 \right)*P_{nonbingedays}+\left( RR_{binge}-1 \right)*P_{bingedays}+1$$

where the risk of injury on average drinking days (RR_average_) was calculated as follows:

$$RR_{average}=P_{dayatrisk}(x)*(RR_{avg\_non-binge}(x)-1)+1$$

and where the risk of injury on binge drinking days (RR_binge_) was calculated as follows:

$$RR_{binge}=P_{dayatrisk}(x)*\left( RR_{binge}\left( x \right)-1 \right)+1$$

where P_dayatrisk_ is the proportion of a day at risk, and RR_binge_ and RR_current_ are the relative risks for injury given an amount of alcohol consumed. P_dayatrisk_ is calculated based on the average rate at which alcohol is metabolized.

To estimate the alchol PAF for mortality from motor vehicle accidents, we multiplied the alcohol PAF for morbidity for motor vehicle accidents by 3/2. Similarly, to estimate the alcohol PAF for mortality due to non-motor vehicle accidents, we multiplied the alcohol PAF for morbidity for non-motor vehicle accidents by 9/4. These multipliers were based on two studies that compared blood alcohol levels of emergency room patients with blood alcohol levels obtained from coroners’ reports of patients who died from an injury [[12](#_ENREF_12), [13](#_ENREF_13)]. The alcohol PAF for motor vehicle accidents for women was calculated by multiplying the alcohol PAF for motor vehicle accidents for men by the product of the *per capita* consumption of alcohol for women divided by the *per capita* consumption of alcohol for men.

*Estimating the alcohol PAFs for harms caused to others*

The alcohol PAFs for injuries caused by an assault by someone who has been drinking were calculated based on recent data reported by [[14](#_ENREF_14)]. These alcohol PAFs were calculated based on the proprtion of all assaults attributable to alcohol consumption in the country of interest (as calculated using the injury PAF method outlined above) as compared to the prortion of all assualts attributable to alcohol consumption in Australia (as obtained from [[14](#_ENREF_14)]). These calcualtions were performed as follows:

$$PAF_{assaultagecountryi}={PAF}_{assaultageAustralia}*(PAF_{assaultcountryi}/PAF_{assaultAustralia})$$

where PAF_assaultagecountryi_ is the alcohol PAF for deaths or injuries caused by assaults for an age group, PAF_assaultcountryi_ is the alcohol PAF for deaths or injuries caused by assaults for an entire country, alcohol PAF_assaultageAustralia_ is the alcohol PAF for deaths or injuries caused by assaults for each specific age group in Australia, and PAF_assaultAustralia_ is the alcohol PAF for deaths or injuries caused by assaults for Australia.

***Modelling of alcohol consumption***

The distribution of alcohol consumption among current drinkers by age and sex was estimated using methodology developed by Rehm and colleagues, and Kehoe and colleagues [[15](#_ENREF_15), [16](#_ENREF_16)]. Using data from 1,001 average daily consumption distributions obtained from over 66 countries, Rehm and colleagues and Kehoe and colleagues observed that average daily alcohol consumption is best modelled, in most instances, using a gamma distribution [[15](#_ENREF_15), [16](#_ENREF_16)]; however, the gamma distribution needs the mean (μ) and standard deviation (σ) parameters to be defined. By regressing more than 851 sex specific means and sex specific standard deviations of average daily alcohol consumption from nationally representative alcohol surveys from 66 countries, Rehm and colleagues and Kehoe and colleagues found that the standard deviation of a population distribution for average daily alcohol consumption could be expressed as a function of the mean as depicted in formulas 2 and 3 [[15](#_ENREF_15), [16](#_ENREF_16)]. These sex specific means had an observed correlation (R^2^) with the standard deviation of 0.94 and thus the mean explained most of the variance in the observed standard deviation [[4](#_ENREF_4)]. Therefore imputation should be should be an accurate method of estimating the standard deviation of the alcohol distribution for most countries. The mean and the standard deviation of the Gamma distribution can be used to characterize the alcohol consumption Gamma distribution by to following formulas.

Reference List

1. Murray CJL, Lopez A: **Global mortality, disability, and the contribution of risk factors: global burden of disease study**. *Lancet* 1997, **349**:1436-1442.

2. Rothman KJ, Greenland S, Lash TL: **Modern Epidemiology, 3rd ed**. Philadelphia, PA: Lippincott Williams & Wilkins; 2008.

3. World Health Organization: **The global burden of disease: 2004 update**. In*.* Geneva, Switzerland: World Health Organization; 2008.

4. Rehm J, Baliunas D, Borges GL, Graham K, Irving HM, Kehoe T, Parry CD, Patra J, Popova L, Poznyak V *et al*: **The relation between different dimensions of alcohol consumption and burden of disease - An overview**. *Addiction* 2010, **105**(5):817-843.

5. Rehm J, Sempos C, Trevisan M: **Average volume of alcohol consumption, patterns of drinking and risk of coronary heart disease - a review**. *Journal of Cardiovascular Risk* 2003, **10**(1):15-20.

6. Puddey IB, Rakic V, Dimmitt SB, Beilin LJ: **Influence of pattern of drinking on cardiovascular disease and cardiovascular risk factors - a review**. *Addiction* 1999, **94**:649-663.

7. Roerecke M, Rehm J: **The cardioprotective association of average alcohol consumption and ischaemic heart disease: a systematic review and meta-analysis**. *Addiction* 2012, **107**(7):1246-1260.

8. Corrao G, Rubbiati L, Bagnardi V, Zambon A, Poikolainen K: **Alcohol and coronary heart disease: a meta-analysis**. *Addiction* 2000, **95**(10):1505-1523.

9. Ronksley PE, Brien SE, Turner BJ, Mukamal KJ, Ghali WA: **Association of alcohol consumption with selected cardiovascular disease outcomes:a systematic review and meta-analysis**. *BMJ* 2011, **342**:d671.

10. Roerecke M, Rehm J: **Irregular heavy drinking occasions and risk of ischemic heart disease: a systematic review and meta-analysis**. *Am J Epidemiol* 2010, **171**(6):633-644.

11. Taylor B, Shield K, Rehm J: **Combining best evidence: a novel method to calculate the alcohol-attributable fraction and its variance for injury mortality**. *BMC public health* 2011, **11**(1):265.

12. Cherpitel C: **Alcohol and casualties: a comparison of emergency room and coroner data**. *Alcohol Alcohol* 1994, **29**:211-218.

13. Cherpitel C: **Alcohol in fatal and nonfatal injuries: a comparison of coroner and emergency room data from the same country**. *Alcoholism: Clinical and Experimental Research* 1996, **20**(2):338-342.

14. Laslett AM, Catalano P, Chikritzhs T, Dale C, Doran C, Ferris J, Jainullabudeen T, Livingston M, Matthews S, Mugavin S *et al*: **The range and magnitude of alcohol's harm to others**. Fitzroy, AU: Turning Point Alcohol & Drug Centre; 2010.

15. Rehm J, Kehoe T, Gmel G, Stinson F, Grant B, Gmel G: **Statistical modeling of volume of alcohol exposure for epidemiological studies of population health: the example of the US**. *Population Health Metrics* 2010, **8**:3.

16. Kehoe T, Gmel G, Jr., Shield K, Gmel G, Sr., Rehm J: **Determining the best population-level alcohol consumption model and its impact on estimates of alcohol-attributable harms**. *Population Health Metrics* 2012, **10**(1):6.
