# Supplementary material for: Russia-specific relative risks and their effects on the estimated alcohol-attributable burden of disease
Source: BMC Public Health. 2015 May 10;15:482. doi: 10.1186/s12889-015-1818-y (PMC4489203; doi:10.1186/s12889-015-1818-y)
Supplement: Additional file 2: — Sources of Relative Risk functions and of Relative Risk function plots. [file 12889_2015_1818_MOESM2_ESM.docx]

**Additional file 2.** Sources of Relative Risk functions and of Relative Risk function plots

| **Table A1.** Sources of Relative Risk functions by ICD-10 Code. | | | | | |
| --- | --- | --- | --- | --- | --- |
| Conditions | | ICD 10 Code | New WHO code | Old WHO code | Sources for Relative Risks |
| Infectious and parasitic diseases | |  |  |  |  |
|  | Tuberculosis | A15-A19 | 3 | IA1 | [[1](#_ENREF_1)]; for causal relationship see: [[2](#_ENREF_2)] |
| Human immunodeficiency virus/ Acquired immune deficiency syndrome | | B20-B24 | 10 | IA3 | [[3](#_ENREF_3), [4](#_ENREF_4)] |
|  |  |  |  |  |  |
| Malignant neoplasm's | |  | 61 | IIA |  |
|  | Mouth and oropharynx cancers | C00-C14 | 62 | IIA1 | [[5](#_ENREF_5)] (based on Relative Risks from [[3](#_ENREF_3)]) |
|  | Esophageal cancer | C15 | 63 | IIA2 |  |
|  | Liver cancer | C22 | 66 | IIA5 |  |
|  | Laryngeal cancer | C32 | 78 |  |  |
|  | Breast cancer | C50 | 70 | IIA9 |  |
|  | Colon cancer | C18 | 65 | IIA4 |  |
|  | Rectal cancer | C20 |  |  |  |
| Diabetes | |  |  |  |  |
|  | Diabetes mellitus | E10-E14 | 80 | IIC | [[6](#_ENREF_6)] |
| Neuro-psychiatric conditions | |  |  |  |  |
|  | Alcoholic psychoses (part of AUD) | F10.0, F10.3-F10.9 | 86 | IIE4 | 100% AAF per definition |
|  | Alcohol abuse (part of AUD) | F10.1 |  |  | 100% AAF per definition |
|  | Alcohol dependence (part of AUD) | F10.2 |  |  | 100% AAF per definition |
|  | Epilepsy | G40-G41 | 97 | IIF3 | [[7](#_ENREF_7)] |
| Cardiovascular disease | |  | 110 | IIH |  |
|  | Hypertensive disease | I10-I15 | 112 | IIH2 | [[8](#_ENREF_8)] |
|  | Ischemic heart disease | I20-I25 | 113 | IIH3 | [[9](#_ENREF_9)] for volume,[[10](#_ENREF_10)] for pattern |
|  | Cardiac arrhythmias | I47-I49 | 116 | IIH6 | [[11](#_ENREF_11)] |
|  | Ischemic strokes | I60-I62 | 114 | IIH4 | [[12](#_ENREF_12)] |
|  | Hemorrhagic and other non-ischemic strokes | I63-I66 | 114 | IIH4 | [[12](#_ENREF_12)] |
| Digestive diseases | |  | 121 | IIJ |  |
|  | Cirrhosis of the liver | K70, K74 | 123 | IIJ2 | [[13](#_ENREF_13)] |
|  | Acute and chronic pancreatitis | K85, K86.1 | 125 |  | [[14](#_ENREF_14)] |
| Respiratory infections | |  |  |  |  |
|  | Pneumonia | J10.0, J11.0, J12-J15, J18 | 39 | IB1 | [[15](#_ENREF_15)] |
| Conditions arising during the prenatal period | |  |  |  |  |
|  | Low birth weight: as defined by the global burden of disease | P05-P07 | 50 | ID1 | [[16](#_ENREF_16)] |
| Unintentional injuries | |  | 152 | IIIA |  |
|  | Motor vehicle accidents | § | 153 | IIIA1 | [[17](#_ENREF_17)] for Relative Risk, methodology adopted from [[18](#_ENREF_18)] * |
|  | Poisonings | X40-X49 | 154 | IIIA2 |  |
|  | Falls | W00-W19 | 155 | IIIA3 |  |
|  | Fires | X00-X09 | 156 | IIIA4 |  |
|  | Drowning | W65-W74 | 157 | IIIA5 |  |
|  | Other Unintentional injuries | †Rest of V-series and W20-W64, W 75-W99, X10-X39, X50-X59, Y40-Y86, Y88, and Y89 | 159 | IIIA7 |  |
| Intentional injuries | |  | 160 | IIIB |  |
|  | Self-inflicted injuries | X60-X84 and Y87.0 | 161 | IIIB1 | [[17](#_ENREF_17)] for Relative Risk, methodology adopted from [[18](#_ENREF_18)] * |
|  | Homicide | X85-Y09, Y87.1 | 162 | IIIB2 |  |
| § V021–V029, V031–V039, V041–V049, V092, V093, V123–V129, V133–V139, V143–V149, V194–V196, V203–V209, V213–V219, V223–V229, V233–V239, V243–V249,V253–V259, V263–V269, V273– V279, V283–V289, V294–V299, V304–V309, V314–V319, V324–V329, V334–V339, V344–V349, V354–V359, V364–V369, V374–V379, V384–V389, V394–V399, V404–V409, V414–V419, V424–V429, V434–V439, V444–V449, V454–V459, V464– V469, V474–V479, V484–V489, V494–V499, V504–V509, V514–V519, V524–V529, V534–V539, V544–V549, V554–V559, V564–V569, V574–V579, V584–V589, V594–V599, V604–V609, V614–V619, V624–V629, V634–V639, V644–V649, V654– V659, V664–V669, V674–V679, V684–V689, V694–V699, V704–V709, V714–V719, V724–V729, V734–V739, V744–V749, V754–V759, V764–V769, V774–V779, V784–V789, V794–V799, V803–V805, V811, V821, V830–V833, V840–V843, V850– V853, V860–V863, V870–V878, V892. †Rest of V = V-series MINUS §.  * The relative risks from [[17](#_ENREF_17)] were adapted from BAC to grams of alcohol consumed. See [[19](#_ENREF_19)] for the formulas in grams of alcohol consumed per drinking occasion. | | | | | |

Reference List

1. Lönnroth K, Williams B, Stadlin S, Jaramillo E, Dye C: **Alcohol use as a risk factor for tuberculosis - a systematic review**. *BMC public health* 2008, **8**:289.

2. Rehm J, Mathers C, Popova S, Thavorncharoensap M, Teerawattananon Y, Patra J: **Global burden of disease and injury and economic cost attributable to alcohol use and alcohol use disorders**. *Lancet* 2009, **373**(9682):2223-2233.

3. Corrao G, Bagnardi V, Zambon A, La Vecchia C: **A meta-analysis of alcohol consumption and the risk of 15 diseases**. *Preventive Medicine* 2004, **38**:613-619.

4. Gmel G, Shield K, Rehm J: **Developing a methodology to derive alcohol-attributable fractions for HIV/AIDS mortality based on alcohol's impact on adherence to antiretroviral medication**. *Population Health Metrics* 2011, **9**(1):5.

5. Baan R, Straif K, Grosse Y, Secretan B, El Ghissassi F, Bouvard V, Alteri A, Cogliano V, On behalf of the W. H. O. International Agency for Research on Cancer monograph working group: **Carcinogenicity of alcoholic beverages**. *Lancet Oncology* 2007, **8**(4):292-293.

6. Baliunas D, Taylor B, Irving H, Roerecke M, Patra J, Mohapatra S, Rehm J: **Alcohol as a risk factor for type 2 diabetes - A systematic review and meta-analysis**. *Diabetes care* 2009, **32**(11):2123-2132.

7. Samokhvalov AV, Irving H, Mohapatra S, Rehm J: **Alcohol consumption, unprovoked seizures and epilepsy: a systematic review and meta-analysis**. *Epilepsia* 2010, **51**(7):1177-1184.

8. Taylor B, Irving HM, Baliunas D, Roerecke M, Patra J, Mohapatra S, Rehm J: **Alcohol and hypertension: gender differences in dose-response relationships determined through systematic review and meta-analysis**. *Addiction* 2009, **104**(12):1981-1990.

9. Roerecke M, Rehm J: **The cardioprotective association of average alcohol consumption and ischaemic heart disease: a systematic review and meta-analysis**. *Addiction* 2012, **107**(7):1246-1260.

10. Roerecke M, Rehm J: **Irregular heavy drinking occasions and risk of ischemic heart disease: a systematic review and meta-analysis**. *Am J Epidemiol* 2010, **171**(6):633-644.

11. Samokhvalov AV, Irving HM, Rehm J: **Alcohol as a risk factor for atrial fibrillation: a systematic review and meta-analysis**. *European Journal of Cardiovascular Prevention & Rehabilitation* 2010, **17**(6):706-712.

12. Patra J, Taylor B, Irving H, Roerecke M, Baliunas D, Mohapatra S, Rehm J: **Alcohol consumption and the risk of morbidity and mortality from different stroke types - a systematic review and meta-analysis**. *BMC public health* 2010, **10**(1):258.

13. Rehm J, Taylor B, Mohapatra S, Irving H, Baliunas D, Patra J, Roerecke M: **Alcohol as a risk factor for liver cirrhosis - a systematic review and meta-analysis**. *Drug Alcohol Rev* 2010, **29**(4):437-445.

14. Irving HM, Samokhvalov A, Rehm J: **Alcohol as a risk factor for pancreatitis. A systematic review and meta-analysis**. *Journal of the Pancreas* 2009, **10**(4):387-392.

15. Samokhvalov AV, Irving HM, Rehm J: **Alcohol consumption as a risk factor for pneumonia: systematic review and meta-analysis**. *Epidemiology and Infection* 2010, **138**(12):1789-1795.

16. Patra J, Bakker R, Irving H, Jaddoe VWV, Malini S, Rehm J: **Dose-response relationship between alcohol consumption before and during pregnancy and the risks of low birthweight, preterm birth and small for gestational age (SGA)-a systematic review and meta-analyses**. *BJOG: International Journal of Obstetrics and Gynaecology* 2011, **118**(12):1411-1421.

17. Taylor B, Irving HM, Kanteres F, Room R, Borges G, Cherpitel C, Greenfield T, Rehm J: **The more you drink, the harder you fall: a systematic review and meta-analysis of how acute alcohol consumption and injury or collision risk increase together**. *Drug Alcohol Depend* 2010, **110**(1-2):108-116.

18. Taylor B, Shield K, Rehm J: **Combining best evidence: a novel method to calculate the alcohol-attributable fraction and its variance for injury mortality**. *BMC public health* 2011, **11**(1):265.

19. Shield KD, Gmel G, Jr., Patra J, Rehm J: **Global burden of injuries attributable to alcohol consumption in 2004: a novel way of calculating the burden of injuries attributable to alcohol consumption**. *Population Health Metrics* 2012, **10**:9.
