# Supplementary material for: Russia-specific relative risks and their effects on the estimated alcohol-attributable burden of disease
Source: BMC Public Health. 2015 May 10;15:482. doi: 10.1186/s12889-015-1818-y (PMC4489203; doi:10.1186/s12889-015-1818-y)
Supplement: Additional file 3: — Comparison of the RR functions from meta-analyses to those obtained from Zaridze et al., [ 14 ]. [file 12889_2015_1818_MOESM3_ESM.docx]

**Additional file 3.** Comparison of the RR functions from meta-analyses to those obtained from Zaridze et al., 2009

| Table A2. Comparison of the RRs from meta-analyses to those obtained from Zaridze et al., 2009 | | | | | | | | | |  |  |  |  |  |  |  |
| --- | --- | --- | --- | --- | --- | --- | --- | --- | --- | --- | --- | --- | --- | --- | --- | --- |
| Disease | Gender | Zaridze et al., 2009 | | | | | |  | Meta-analyses used in the 2010 GBD study | | | | | |  |  |
|  |  | 12.68 to <25.36 g /day | | 25.36 to <76.08 g /day | | ≥ 76.08 g /day | |  | 12.68 to <25.36 g /day | | 25.36 to <76.08 g /day | | ≥ 76.08 g /day | | Former drinkers | |
|  |  | PE | 95% CI | PE | 95% CI | PE | 95% CI |  | PE | 95% CI | PE | 95% CI | PE | 95% CI | PE | 95% CI |
| Acute and chronic pancreatitis | Men | 1.43 | (1.04 to 1.96) | 2.07 | (1.53 to 2.80) | 6.69 | (4.98 to 8.99) |  | 1.06 | (1.04 to 1.07) | 1.46 | (1.34 to 1.63) | 7.35 | (4.57 to 13.32) | 1.21 | (1.10 to 1.33) |
|  | Women | 1.09 | (0.70 to 1.69) | 5.01 | (3.45 to 7.27) | 19.26 | (13.64 to 27.19) |  | 1.06 | (1.04 to 1.07) | 1.38 | (1.27 to 1.54) | 5.87 | (3.68 to 10.36) | 1.44 | (1.28 to 1.61) |
| Pneumonia | Men | 0.95 | (0.82 to 1.10) | 1.92 | (1.51 to 2.43) | 3.29 | (2.55 to 4.24) |  | 1.09 | (1.02 to 1.18) | 1.25 | (1.05 to 1.51) | 1.66 | (1.12 to 2.55) | 1.21 | (1.10 to 1.33) |
|  | Women | 2.10 | (1.74 to 2.53) | 3.21 | (2.56 to 4.02) | 3.42 | (2.64 to 4.43) |  | 1.09 | (1.02 to 1.17) | 1.23 | (1.04 to 1.47) | 1.61 | (1.09 to 2.42) | 1.44 | (1.28 to 1.61) |
| Tuberculosis | Men | 1.01 | (0.83 to 1.23) | 1.97 | (1.64 to 2.37) | 4.14 | (3.44 to 4.99) |  | 1.00 | (1.00 to 1.00) | 2.18 | (1.80 to 2.76) | 2.96 | (2.30 to 3.87) | 1.21 | (1.10 to 1.33) |
|  | Women | 0.93 | (0.64 to 1.35) | 4.06 | (2.97 to 5.56) | 5.32 | (3.70 to 7.65) |  | 1.00 | (1.00 to 1.00) | 1.97 | (1.62 to 2.55) | 2.96 | (2.28 to 3.83) | 1.44 | (1.28 to 1.61) |
| Liver cirrhosis | Men | 0.92 | (0.76 to 1.11) | 1.77 | (1.47 to 2.13) | 6.21 | (5.15 to 7.48) |  | 1.70 | (1.59 to 1.83) | 4.01 | (3.31 to 4.95) | 23.05 | (14.99 to 36.91) | 1.31 | (0.67 to 2.57) |
|  | Women | 2.50 | (2.09 to 2.99) | 7.07 | (5.87 to 8.52) | 12.08 | (9.83 to 14.84) |  | 4.07 | (3.39 to 4.92) | 8.80 | (6.56 to 12.07) | 26.84 | (17.52 to 43.22) | 6.5 | (2.21 to 19.10) |
| Stroke | Men | 1.06 | (0.96 to 1.17) | 1.14 | (1.03 to 1.26) | 1.28 | (1.15 to 1.43) |  | 0.99 | (0.95 to 1.09) | 1.18 | (1.11 to 1.34) | 1.71 | (1.50 to 2.11) | 1.33 | (0.91 to 1.95) |
|  | Women | 1.38 | (1.27 to 1.50) | 1.36 | (1.19 to 1.55) | 1.62 | (1.37 to 1.92) |  | 0.93 | (0.84 to 1.22) | 1.35 | (1.12 to 1.97) | 4.09 | (2.59 to 6.98) | 1.15 | (0.70 to 1.89) |

The categorical RR estimates for acute and chronic pancreatitis, pneumonia, tuberculosis, liver cirrhosis and stroke were calculated using the number of deaths by age, the estimated attributable fraction for the alcohol consumption category and the prevalence of people in each alcohol consumption category. Estimation of the categorical RRs ischemic heart disease, and injuries (motor vehicle accidents, self-inflicted injuries, homicide/assault, injuries of undetermined intent, and other unintentional injuries) were not possible due to differences in how the functions were modeled (the RR functions for ischemic heart disease and injuries also take into account binge alcohol consumption).

* RRs for injuries, IHD and strokes are not directly comparable
