# Supplementary material for: Russia-specific relative risks and their effects on the estimated alcohol-attributable burden of disease
Source: BMC Public Health. 2015 May 10;15:482. doi: 10.1186/s12889-015-1818-y (PMC4489203; doi:10.1186/s12889-015-1818-y)
Supplement: Additional file 4: — YLL and YLD attributable to alcohol consumption in Russia in 2012 using Russia-specific alcohol RR functions and general population alcohol RR functions for people 0 to 64 years of age. [file 12889_2015_1818_MOESM4_ESM.docx]

**Additional file 4.** YLL and YLD lost attributable to alcohol consumption in Russia in 2012 using Russia-specific alcohol RR functions and general population alcohol RR functions for people 0 to 64 years of age

| **Table A3a.** Burden of YLLs attributable to alcohol consumption in Russia for people 0 to 64 years of age in 2012 based on Russia-specific alcohol RR functions | | | | | | | | | | |
| --- | --- | --- | --- | --- | --- | --- | --- | --- | --- | --- |
| Cause* | | | **Women** | |  | **Men** | |  | **Total** | |
|  |  |  | YLL | % of all YLL AA |  | YLL | % of all YLL AA |  | YLL | % of all YLL AA |
| Total | | | 2,910,000 | 30.20% |  | 7,113,000 | 29.50% |  | 10,023,000 | 29.7% |
|  | Communicable, maternal, neonatal and nutritional disorders | | 170,700 | 11.80% |  | 572,300 | 15.70% |  | 743,000 | 14.6% |
|  |  | HIV/AIDS and tuberculosis | 79,100 | 9.00% |  | 390,300 | 14.60% |  | 469,400 | 13.2% |
|  |  | Lower respiratory infections | 83,900 | 36.10% |  | 170,800 | 32.30% |  | 254,700 | 33.4% |
|  |  | Neonatal conditions | 7,700 | 3.30% |  | 11,200 | 3.40% |  | 18,900 | 3.3% |
|  | Non-communicable diseases | | 1,977,900 | 29.30% |  | 3,343,200 | 22.90% |  | 5,321,100 | 24.9% |
|  |  | Neoplasms | 141,500 | 6.80% |  | 288,300 | 10.00% |  | 429,800 | 8.6% |
|  |  | Diabetes mellitus | -10,400 | -13.80% |  | -400 | -0.50% |  | -10,800 | -7.5% |
|  |  | Cirrhosis of the liver | 489,900 | 76.10% |  | 505,900 | 49.80% |  | 995,800 | 60.0% |
|  |  | Digestive diseases (except cirrhosis | 40,000 | 16.70% |  | 144,300 | 19.20% |  | 184,300 | 18.6% |
|  |  | Neurological disorders | 4,600 | 4.80% |  | 10,300 | 8.10% |  | 14,900 | 6.7% |
|  |  | Mental and behavioral disorders | 147,200 | 61.30% |  | 640,000 | 65.90% |  | 787,200 | 65.0% |
|  |  | Cardiovascular and circulatory diseases | 1,165,100 | 41.40% |  | 1,754,700 | 22.10% |  | 2,919,800 | 27.2% |
|  | Injuries | | 761,100 | 53.40% |  | 3,197,900 | 54.50% |  | 3,959,000 | 54.3% |
|  |  | Transport injuries | 117,100 | 41.60% |  | 460,700 | 47.80% |  | 577,800 | 46.4% |
|  |  | Unintentional non-transport injuries** | 418,000 | 54.30% |  | 1,684,300 | 54.40% |  | 2,102,300 | 54.4% |
|  |  | Self harm and personal violence*** | 226,100 | 61.40% |  | 1,052,900 | 60.10% |  | 1,279,000 | 60.3% |

* See additional file number 2 for ICD categories included in each cause

** Includes poisonings, falls, fires, drowning and other unintentional injuries

*** Includes self-inflicted injuries and homicide

| **Table A3b.** Burden of YLDs attributable to alcohol consumption in Russia for people 0 to 64 years of age in 2012 based on Russia-specific alcohol RR functions | | | | | | | | | | |
| --- | --- | --- | --- | --- | --- | --- | --- | --- | --- | --- |
| Cause* | | | **Women** | |  | **Men** | |  | **Total** | |
|  |  |  | YLD | % of all YLD AA |  | YLD | % of all YLD AA |  | YLD | % of all YLD AA |
| Total | | | 761,000 | 10.70% |  | 2,511,000 | 33.50% |  | 3,272,000 | 22.4% |
|  | Communicable, maternal, neonatal and nutritional disorders | | 12,500 | 1.70% |  | 47,700 | 6.90% |  | 60,200 | 4.3% |
|  |  | HIV/AIDS and tuberculosis | 8,500 | 11.80% |  | 44,300 | 20.30% |  | 52,800 | 18.2% |
|  |  | Lower respiratory infections | 2,800 | 4.20% |  | 2,100 | 3.00% |  | 4,900 | 3.5% |
|  |  | Neonatal conditions | 1,200 | 2.40% |  | 1,400 | 2.20% |  | 2,600 | 2.3% |
|  | Non-communicable diseases | | 563,200 | 9.30% |  | 2,144,400 | 34.50% |  | 2,707,600 | 22.0% |
|  |  | Neoplasms | 3,900 | 9.50% |  | 3,500 | 11.00% |  | 7,400 | 10.2% |
|  |  | Diabetes mellitus | -35,200 | -13.70% |  | -1,100 | -0.50% |  | -36,300 | -7.9% |
|  |  | Cirrhosis of the liver | 5,000 | 75.80% |  | 4,100 | 49.90% |  | 9,100 | 61.4% |
|  |  | Digestive diseases (except cirrhosis | 3,600 | 7.40% |  | 8,600 | 15.50% |  | 12,200 | 11.7% |
|  |  | Neurological disorders | 12,000 | 2.80% |  | 22,100 | 8.40% |  | 34,100 | 4.9% |
|  |  | Mental and behavioral disorders | 490,300 | 18.70% |  | 2,058,200 | 57.90% |  | 2,548,500 | 41.3% |
|  |  | Cardiovascular and circulatory diseases | 83,700 | 36.30% |  | 49,000 | 19.20% |  | 132,700 | 27.3% |
|  | Injuries | | 184,900 | 54.00% |  | 319,200 | 53.40% |  | 504,100 | 53.6% |
|  |  | Transport injuries | 40,100 | 40.30% |  | 91,800 | 46.40% |  | 131,900 | 44.4% |
|  |  | Unintentional non-transport injuries** | 137,800 | 59.40% |  | 206,300 | 56.30% |  | 344,100 | 57.5% |
|  |  | Self harm and personal violence*** | 7,100 | 64.90% |  | 21,100 | 62.90% |  | 28,200 | 63.4% |

* See additional file number 2 for ICD categories included in each cause

** Includes poisonings, falls, fires, drowning and other unintentional injuries

*** Includes self-inflicted injuries and homicide
