# Supplementary material for: Russia-specific relative risks and their effects on the estimated alcohol-attributable burden of disease
Source: BMC Public Health. 2015 May 10;15:482. doi: 10.1186/s12889-015-1818-y (PMC4489203; doi:10.1186/s12889-015-1818-y)
Supplement: Additional file 5: — Deaths, YLL, YLD and DALYs lost attributable to alcohol consumption using Russia-specific alcohol RR functions and general population alcohol RR functions for people of all ages. [file 12889_2015_1818_MOESM5_ESM.docx]

**Web appendix 5.** Deaths, YLL, YLD and DALYs attributable to alcohol consumption using Russia specific RR functions and general population RR functions for people of all ages

**Table A4.** Deaths, YLL, YLD and DALYs attributable to alcohol consumption using Russia specific RR functions and general population RR functions for people of all ages

|  |  |  | Women | | | |  | Men | | | |
| --- | --- | --- | --- | --- | --- | --- | --- | --- | --- | --- | --- |
|  | **Disease, condition or injury** | | Deaths | YLL | YLD | DALYs |  | Deaths | YLL | YLD | DALYs |
| **Russia specific RR** | | |  |  |  |  |  |  |  |  |  |
|  | Communicable, maternal, neonatal and nutritional disorders | |  |  |  |  |  |  |  |  |  |
|  |  | Lower respiratory infection | 4,810 | 124,800 | 6,200 | 130,900 |  | 7,420 | 221,500 | 3,200 | 225,000 |
|  | Non-communicable diseases | |  |  |  |  |  |  |  |  |  |
|  |  | Ischemic heart disease | 252,270 | 3,980,800 | 162,000 | 4,142,800 |  | 105,640 | 2,579,900 | 66,400 | 2,646,000 |
|  |  | Ischemic strokes | 50,140 | 706,800 | 15,800 | 722,700 |  | 16,830 | 329,700 | 7,400 | 337,000 |
|  |  | Hemorrhagic and other non-ischemic strokes | 10,980 | 249,300 | 2,300 | 251,600 |  | 6,410 | 188,700 | 1,500 | 190,000 |
|  |  | Cirrhosis of the liver | 16,180 | 570,100 | 6,800 | 577,000 |  | 13,960 | 550,500 | 4,800 | 555,000 |
|  |  | Acute and chronic pancreatitis | 1,570 | 49,600 | 4,400 | 54,000 |  | 4,150 | 162,300 | 9,500 | 172,000 |
|  | Injuries | |  |  |  |  |  |  |  |  |  |
|  |  | Transport injuries | 2,890 | 131,200 | 49,700 | 180,900 |  | 9,000 | 475,700 | 103,600 | 579,000 |
|  |  | Unintentional injuries (other than transport injuries) | 12,620 | 475,600 | 208,500 | 684,000 |  | 41,100 | 1,795,300 | 271,200 | 2,066,000 |
|  |  | Self harm and personal violence | 6,240 | 256,600 | 8,200 | 264,800 |  | 22,980 | 1,108,800 | 22,600 | 1,131,000 |
|  | Total | | 371,200 | 7,007,000 | 967,000 | 7,975,000 |  | 269,900 | 8,990,000 | 2,699,000 | 11,688,000 |
| **Non-Russian specific RRs** | | |  |  |  |  |  |  |  |  |  |
|  | Communicable, maternal, neonatal and nutritional disorders | |  |  |  |  |  |  |  |  |  |
|  |  | Lower respiratory infection | 880 | 24,600 | 1,100 | 25,700 |  | 3,150 | 94,700 | 1,400 | 96,100 |
|  | Non-communicable diseases | |  |  |  |  |  |  |  |  |  |
|  |  | Ischemic heart disease | 9,570 | 131,500 | (40,100) | 91,400 |  | (9,250) | (273,800) | (21,200) | (295,000) |
|  |  | Ischemic strokes | (5,980) | (84,600) | (3,800) | (88,400) |  | 4,180 | 94,600 | 2,000 | 96,600 |
|  |  | Hemorrhagic and other non-ischemic strokes | 8,110 | 214,300 | (600) | 213,700 |  | 9,910 | 301,800 | 2,800 | 304,600 |
|  |  | Cirrhosis of the liver | 13,320 | 479,700 | 6,600 | 486,300 |  | 22,670 | 888,100 | 5,200 | 893,300 |
|  |  | Acute and chronic pancreatitis | 500 | 17,100 | 1,500 | 18,600 |  | 3,680 | 146,200 | 8,600 | 154,800 |
|  | Injuries | |  |  |  |  |  |  |  |  |  |
|  |  | Transport injuries | 1,870 | 83,700 | 24,900 | 108,600 |  | 16,470 | 858,000 | 144,100 | 1,002,200 |
|  |  | Unintentional injuries (other than transport injuries) | 3,730 | 154,000 | 26,400 | 180,400 |  | 45,840 | 2,076,500 | 129,100 | 2,205,600 |
|  |  | Self harm and personal violence | 1,630 | 79,200 | 1,000 | 80,300 |  | 24,050 | 1,224,500 | 10,400 | 1,234,900 |
|  | Total | | 47,100 | 1,562,000 | 521,000 | 2,083,000 |  | 163,100 | 6,988,000 | 2,491,000 | 9,479,000 |
